# A Case of Metastatic Urothelial Carcinoma-Associated Acute Esophageal Necrosis

**DOI:** 10.7759/cureus.62521

**Published:** 2024-06-17

**Authors:** Zhongqian Lin, Aruni Rahman

**Affiliations:** 1 Internal Medicine, New York Presbyterian Brooklyn Methodist Hospital, Brooklyn, USA

**Keywords:** high-grade urothelial carcinoma, esophagogastroduodenoscopy (egd), severe hematemesis, hospice and palliative care, esophageal necrosis

## Abstract

Acute esophageal necrosis is a rare syndrome with endoscopic findings of a diffuse circumferential pattern of black mucosa. Although underlying pathogenesis is unclear, it is known to have associations with malignancy. We present a rare case of a patient with a history of metastatic urothelial carcinoma who was found to have acute esophageal necrosis.

## Introduction

Acute esophageal necrosis is a rare finding, described as black mucosa, in a diffuse circumferential pattern. Most commonly, it affects the distal third portion of the esophagus where the esophagus is more hypovascular and does not pass the gastroesophageal junction. The exact pathogenesis of acute esophageal is still unknown but likely related to gastric outlet obstruction or ischemia [1,2]. There were case reports of possible associations such as with malignancy, infection, antibiotic use, diabetic ketoacidosis, or prolonged vomiting. The symptoms typically present as hematemesis and/or melena [3]. We present a case of a male with high-grade metastatic urothelial carcinoma who presented with coffee-ground emesis and melena and was found to have acute esophageal necrosis on upper endoscopy.

## Case presentation

**Figure 1:**
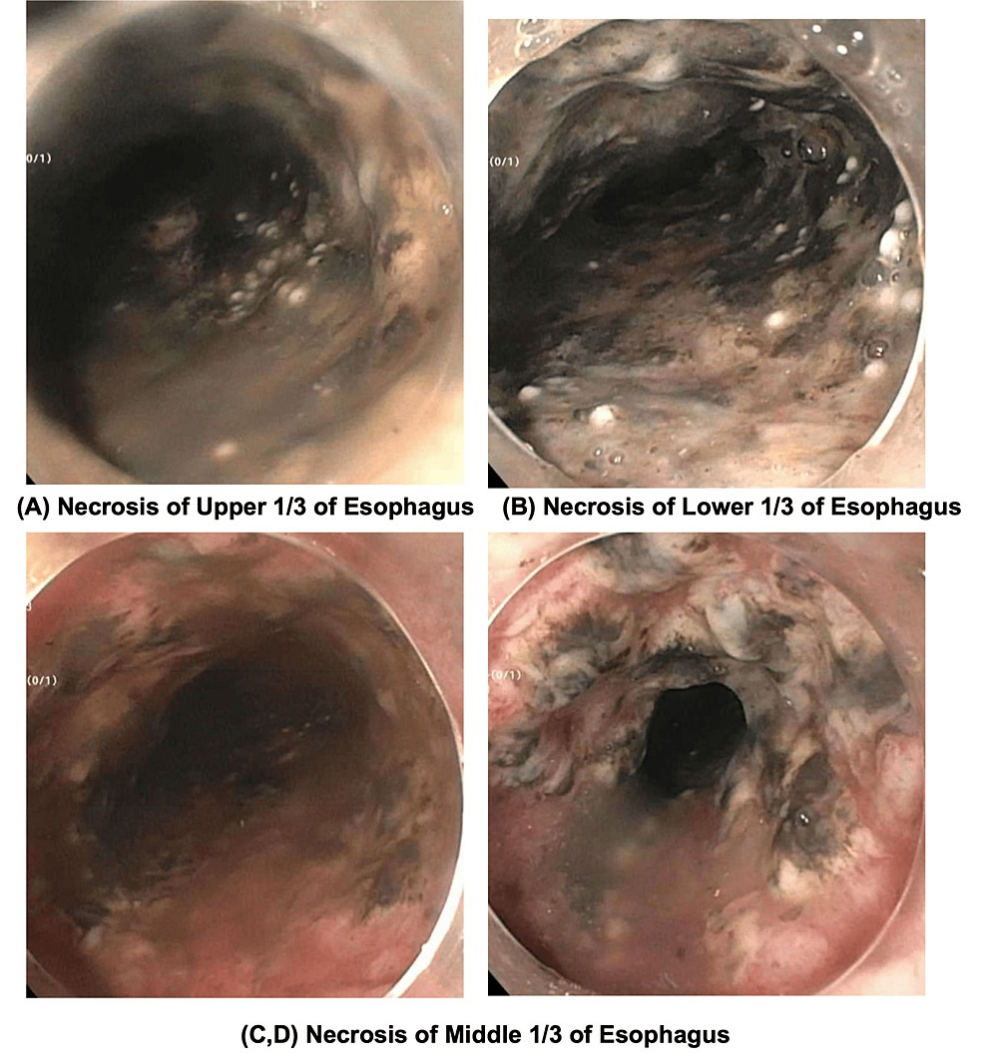
Upper endoscopic findings of esophageal necrosis

A 64-year-old male with a history of high-grade urothelial carcinoma with diffuse bone metastasis on pembrolizumab and status post-bilateral percutaneous nephrostomy (PCN) tubes presented initially with suprapubic and right flank pain. He was subsequently admitted for the management of a complicated urinary tract infection (UTI) and exchange of a malfunctioned right PCN tube. The patient was noted to have dysphagia and decreased appetite and at the time the symptoms contributed to the nature of his malignancy. However, he developed an episode of coffee-ground emesis and melena as well as hemodynamic instability with systolic blood pressure in the range of 60s and more than 2 g/dL of hemoglobin drop from his baseline, although CT angiography is negative for any active extravasation of blood. After the initial resuscitation, the patient underwent urgent esophagogastroduodenoscopy (EGD), which demonstrated diffuse severe mucosal changes characterized by black esophagus as well as necrotic mucosa found in the entire esophagus from 25 cm to 39 cm (Figure 1). However, the esophageal biopsy was not performed as the patient redeveloped hemodynamical instability during the endoscopy. In light of the patient's compromised functional status, characterized by confinement to bed and a suboptimal nutritional state, coupled with the family's prior encounter with cancer-related challenges, they have judiciously chosen to pursue hospice and comfort care for the patient.

## Discussion

Acute esophageal necrosis is a rare diagnosis, and as clinicians, it is not often immediately associated with malignancy. There were only sporadic reports regarding similar clinical vignettes, such as a case report of acute esophageal necrosis in prostate cancer post-chemotherapy [4].

Interestingly, the entire esophagus was necrotic from the upper third to the lower third portions, different from the common distribution of necrosis, which is in the distal part of the esophagus. Although there are currently no clear guidelines for the management of acute esophageal necrosis, it is understood to be irreversible and has the ability to have severe complications such as esophageal perforation, stricture, or bleeding.

Due to the permanent nature of esophageal necrosis, early recognition of symptoms is critical to guide patient care toward approaches focused on comfort over interventions. Heightened vigilance among physicians regarding this association and timely identification of symptoms could enable prompt pharmacological, endoscopic, or surgical options aimed at managing pain and nutrition challenges. Although the prognosis remains poor, earlier implementation of palliative-focused care, prompted by timely recognition of esophageal necrosis, can enable improved quality of life throughout the terminal phase of illness.

## Conclusions

Acute esophageal necrosis, though rare, can manifest with severe clinical symptoms and typically signifies a poor overall prognosis. It is crucial for clinicians to promptly recognize this condition, particularly in patients with potentially associated factors such as high-grade urothelial carcinoma, as seen in this case. Early diagnosis is essential to ensure that the care provided aligns with the patient's goals and optimizes clinical outcomes.
